# Factors associated with sepsis development in 606 Spanish adult patients with cellulitis

**DOI:** 10.1186/s12879-020-4915-1

**Published:** 2020-03-12

**Authors:** J. Collazos, B. de la Fuente, J. de la Fuente, A. García, H. Gómez, C. Menéndez, H. Enríquez, P. Sánchez, M. Alonso, I. López-Cruz, M. Martín-Regidor, A. Martínez-Alonso, J. Guerra, A. Artero, M. Blanes, V. Asensi

**Affiliations:** 1 Infectious Diseases Unit, Hospital de Galdácano, Vizcaya, Spain; 2 Infectious Diseases Unit, Hospital de Cabueñes, Gijón, Spain; 3 Internal Medicine Service, Hospital de Povisa, Vigo, Spain; 4 Infectious Diseases Unit, Hospital Universitario Central de Asturias, Oviedo, Spain; 5 Internal Medicine Service, Hospital Dr Peset, Valencia, Spain; 6 Internal Medicine Service, Complejo Hospitalario de León, León, Spain; 7 Infectious Diseases Unit, Hospital La Fe, Valencia, Spain; 8 Group of Translational Research in Infectious Diseases, Instituto de Investigación Sanitaria del Principado de Asturias (ISPA), Oviedo, Spain

**Keywords:** Cellulitis, Sepsis, Outcome, Antibiotics, Surgery

## Abstract

**Background:**

Cellulitis, a frequent cause of admission of adult patients to medical wards, occasionally evolves to sepsis. In this study we analyze the factors related to sepsis development.

**Methods:**

Prospective and observational study of 606 adult patients with cellulitis admitted to several Spanish hospitals. Comorbidities, microbiological, clinical, lab, diagnostic, and treatment data were analyzed. Sepsis was diagnosed according to the criteria of the 2016 International Sepsis Definitions Conference. Multiple logistic regression modelling was performed to determine the variables independently associated with sepsis development.

**Results:**

Mean age was 63.4 years and 51.8% were men. Overall 65 (10.7%) patients developed sepsis, 7 (10.8%) of whom died, but only 4 (6.2%) due to cellulitis. Drawing of blood (*P* < 0.0001) or any (P < 0.0001) culture, and identification of the agent (*P* = 0.005) were more likely among patients with sepsis. These patients had also a longer duration of symptoms (*P* = 0.04), higher temperature (*P* = 0.03), more extensive cellulitis (*P* = 0.02), higher leukocyte (*P* < 0.0001) and neutrophil (*P* < 0.0001) counts, serum creatinine (*P* = 0.001), and CRP (*P* = 0.008) than patients without sepsis. Regarding therapy, patients with sepsis were more likely to undergo changes in the initial antimicrobial regimen (*P* < 0.0001), received more antimicrobials (*P* < 0.0001), received longer intravenous treatment (*P* = 0.03), and underwent surgery more commonly (*P* = 0.01) than patients without sepsis. Leukocyte counts (*P* = 0.002), serum creatinine (*P* = 0.003), drawing of blood cultures (*P* = 0.004), change of the initial antimicrobial regimen (*P* = 0.007) and length of cellulitis (*P* = 0.009) were independently associated with sepsis development in the multivariate analysis.

**Conclusions:**

Increased blood leukocytes and serum creatinine, blood culture drawn, modification of the initial antimicrobial regimen, and maximum length of cellulitis were associated with sepsis in these patients.

## Background

Cellulitis, a common type of skin and soft tissue infection (SSTI), is a frequent cause of admission of adult patients to medical wards in all age ranges. In a recent large study using U.S. health insurance data over 2.3 million episodes of SSTI in patients aged 0–64 years were identified between 2005 and 2010 [1]. Although 95% of them were treated in the ambulatory setting, 209,944 patients still required hospital admission. Overall the incidence of SSTIs represented about 48 episodes per 1000 person years in this study, an incidence that was twice that of urinary tract infections and tenfold of that of pneumonia [1]. In the Netherlands the annual incidence of cellulitis was estimated to be 22 per 1000 inhabitants, and approximately 7% of patients with cellulitis are hospitalized [2, 3].

Cellulitis can lead to bacteremia and ultimately trigger sepsis development. The frequency of bacteremia in patients with cellulitis ranges from 2 to 21.3% among patients for whom blood cultures were available, depending on the study, setting and typology of the cases analyzed [4–16]. Taking into account that most cases of cellulitis are treated in the ambulatory setting and that blood cultures are not obtained in many hospitalized patients with less severe cellulitis, populations in which expectedly bacteremia occurs more infrequently, the overall prevalence of positive blood cultures considering the full spectrum of cellulitis would be even lower.

This expected low yield of blood cultures and the fact that blood culture results did not affect treatment in complicated cellulitis led the Infectious Diseases Society of America (IDSA) to not recommending in the current guidelines the routine performance of blood cultures in patients with cellulitis and erysipelas, except for specific populations [12, 17].

However, other studies have shown that older age, male sex, diabetes, infected devices, alcohol abuse, cirrhosis, lymphedema and diverse other comorbidities increase the probability of bacteremia and, therefore, the performance of blood cultures in these patients would be advisable [9–16, 18].

To identify the factors predisposing to bacteremia in cellulitis is an area of utmost interest for the clinician taking into account that sepsis constitutes a serious and potentially deadly complication of this SSTI infection. Although a number of studies describing the prevalence and factors related to bacteremia in cellulitis have been published, it is remarkable that after an extensive literature search we failed to find any study focused on the analysis and identification of the factors predisposing to or associated with sepsis. A recent Dutch study compared the clinical characteristics and outcome of patients with cellulitis (*n* = 23) and necrotizing fasciitis (*n* = 31) admitted to an intensive care unit [7]. In this study, patients with cellulitis were less critically ill, had more chronic comorbidities and similar short and long-term mortality than patients with necrotizing fasciitis. However, the study did not analyze the factors leading to or associated with sepsis in these patients.

All these clinical and healthcare circumstances and the lack of studies emphasize the value of identifying the factors leading to sepsis, for prevention purposes and for improving the management and outcome of SSTIs. Consequently, the availability of studies that analyze the different parameters that could be associated with the development of sepsis in patients with cellulitis is highly desirable.

The aim of this prospective and multicenter study was to detect the parameters related to sepsis in a large sample of patients hospitalized because of cellulitis. With this purpose all patients underwent a comprehensive evaluation from a demographic, clinical, bacteriological, laboratory, radiological and therapeutic point of view.

## Methods

The study population was composed of adult patients (≥18 years) admitted from 1 January 2016 to 30 June 2017 because of cellulitis to the Internal Medicine departments of the following Spanish institutions: Hospital de Cabueñes, Gijón, Hospital Universitario Central de Asturias (HUCA), Oviedo, Hospital de Povisa, Vigo, Hospital Dr. Peset, Valencia, Hospital La Fe, Valencia, and Complejo Hospitalario de León. Cellulitis was diagnosed by means of the usual clinical criteria [3, 17, 19–22], with the additional support of bacteriological and imaging data. A follow-up of 30 days after discharge was scheduled to verify the outcome of the infection. The diagnosis of sepsis was made according to the 1992 sepsis definitions [23] and the 2016 revision [24].

A large number of data was prospectively recorded for each cellulitis case, including demographic, location, predisposing factors, clinical, lab, imaging, blood/pus culture, hospitalization, outcome and treatment parameters, and evaluated in accordance with the development or not of sepsis. As the results of cultures from superficial samples, such as those of ulcers, abrasions or exudates, may be not reliable because of external contamination, only cultures obtained from purulent collections were considered in order to analyze the microbiological results at the cellulitis foci.

All patients underwent standard care and management, and no specific diagnostic or therapeutic procedures were utilized for this study. Also, the patients’ identification data were concealed to the investigators not involved in their care. Consequently, no written informed consent for study enrollment was required, and a waiver was granted by the Research Ethics Committee of the Principality of Asturias.

### Statistical analysis

Descriptive data are reported as percentage for categorical and mean (95% CI) for continuous variables. The latter variables were transformed into their natural logarithmic values for the sole purpose of statistical analysis, because the original values did not follow a Gaussian distribution. The transformed values were back-transformed after analysis to be reported in the original units. Categorical variables were compared with the chi-square or the Fisher’s exact tests, as appropriate, whereas the *t*-test was used for the comparison of continuous variables. Multivariate analysis was performed by means of a stepwise, conditional logistic regression to detect the parameters that were associated with the development of sepsis. Statistical tests were carried out with the SPSS (v.22) software (IBM Corporation, Armonk, NY). The level of statistical significance was established at *P* < 0.05 for a two-sided test.

### Results

A total of 606 adult patients with cellulitis, 65 (10.7%) with and 541 without sepsis (89.3%), admitted to the participating hospitals from January 2016 to June 2017 were included. Table 1 shows the demographic features, as well as the main comorbidities and potential predisposing factors for the development of cellulitis and sepsis.

**Table 1:** Demography and predisposing factors of patients with cellulitis

	All ***n*** = 606	No sepsis (***n*** = 541)	Sepsis (***n*** = 65)	***P*** value
Demography & anthropometry				
Gender				
Male	314 (51.8%)	276 (51.0%)	38 (58.5%)	0.3
Female	292 (48.2%)	265 (49.0%)	27 (41.5%)	
Age (years)	63.43 (61.85–65.05)	63.42 (61.76–65.12)	63.53 (58.29–69.22)	1
Body mass index (kg/m^2^)				
(*n* = 350)	30.00 (29.25–30-77)	30.05 (29.28–30.83)	29.58 (26.73–32.73)	0.7
Predisposing factors / comorbidities				
Prior cellulitis				
Yes	156 (25.7%)	139 (25.7%)	17 (26.2%)	0.9
No	450 (74.3%)	402 (74.3%)	48 (73.8%)	
Episodes of prior cellulitis				
(Only if prior cellulitis)	1.72 (1.56–1.90)	1.71 (1.54–1.89)	1.86 (1.32–2.61)	0.6
Episodes of prior cellulitis				
0	450 (74.3%)	402 (74.3%)	48 (73.8%)	0.4
1	79 (13.0%)	71 (13.1%)	8 (12.3%)	
2	27 (4.5%)	24 (4.4%)	3 (4.6%)	
3	26 (4.3%)	25 (4.6%)	1 (1.5%)	
4 or more	24 (4.0%)	19 (3.5%)	5 (7.7%)	
Location of prior cellulitis				
Same location	143 (91.7%)	129 (92.8%)	14 (82.4%)	0.15
Other locations	13 (8.3%)	10 (7.2%)	3 (17.6%)	
Prior wounds				
Yes	332 (54.8%)	295 (54.5%)	37 (56.9%)	0.7
No	274 (45.2%)	54.5% (45.5%)	28 (43.1%)	
Type of wound				
None	274 (45.2%)	246 (45.5%)	28 (43.1%)	0.8
Skin ulcer	110 (18.2%)	96 (17.7%)	14 (21.5%)	
Non-surgical trauma	108 (17.8%)	94 (17.4%)	14 (21.5%)	
Surgical	34 (5.6%)	32 (5.9%)	2 (3.1%)	
Animal bite	12 (2.0%)	12 (2.2%)	0 (0.0%)	
Injection	11 (1.8%)	9 (1.7%)	2 (3.1%)	
Arthropod bite	11 (1.8%)	10 (1.8%)	1 (1.5%)	
Others	46 (7.6%)	42 (7.8%)	4 (6.2%)	
Prior skin lesions				
Yes	182 (30.0%)	164 (30.3%)	18 (27.7%)	0.7
No	424 (70.0%)	377 (69.7%)	47 (72.3%)	
Diabetes				
Yes	153 (25.2%)	131 (24.2%)	22 (33.8%)	0.09
No	453 (74.8%)	410 (75.8%)	43 (66.2%)	
Varices				
Yes	124 (20.5%)	112 (20.7%)	12(18.5%)	0.7
No	482 (79.5%)	429 (79.3%)	53 (81.5%)	
Prior deep venous thrombosis				
Yes	23 (3.8%)	21 (3.9%)	2 (3.1%)	0.9
No	583 (95.9%)	520 (96.1%)	63 (96.9%)	
Edema / lymphedema				
Yes	168 (27.7%)	151 (27.9%)	17 (26.2%)	0.8
No	438 (72.3%)	390 (72.1%)	48 (73.8%)	
Heart failure				
Yes	101 (16.7%)	90 (16.6%)	11 (16.9%)	1
No	505 (83.3%)	451 (83.4%)	54 (83.1%)	
Obesity				
Yes	229 (37.8%)	208 (38.4%)	21 (32.3%)	0.3
No	377 (62.2%)	333 (61.6%)	44 (67.7%)	
Immunosuppression				
Yes	70 (11.6%)	62 (11.5%)	8 (12.3%)	0.8
No	536 (88.4%)	479 (88.5%)	57 (87.7%)	
Intravenous drug use				
Yes	7 (1.2%)	7 (1.3%)	0 (0.0%)	1
No	599 (98.8%)	534 (98.7%)	65 (100%)	
HIV infection				
Yes	10 (1.7%)	10 (1.8%)	0 (0.0%)	0.6
No	596 (98.3%)	531 (98.2%)	65 (100%)	
Other comorbidities				
Yes	452 (74.6%)	398 (73.6%)	54 (83.1%)	0.096
No	154 (25.4%)	143 (26.4%)	11 (16.9%)	

Values are expressed as mean (95% CI) or % as appropriate

Mean age was 63.3 years and 51.8% were men. Overall, 65/606 (10.7%) patients developed sepsis. There were no significant differences when comparing demographic or predisposing factors between patients with and without sepsis, although there was a trend towards an association between diabetes (*P* = 0.09) and diverse other comorbidities (*P* = 0.096) with sepsis.

The clinical, laboratory, imaging and hospitalization parameters are shown in Table 2. Patients with sepsis had a shorter duration of symptoms (*P* = 0.04), higher temperature (*P* = 0.03), and more extensive cellulitis (*P* = 0.02). From a laboratory perspective, patients with sepsis had higher leukocyte (*P* < 0.0001) and neutrophil (*P* < 0.0001) counts, serum creatinine (*P* = 0.001), and CRP (*P* = 0.008) than patients without sepsis.

**Table 2:** Clinical, laboratory, imaging and hospitalization parameters associated with sepsis in patients with cellulitis

	All ***n*** = 606	No sepsis (***n*** = 541)	Sepsis (***n*** = 65)	***P*** value
Clinical and topographical aspects				
Days of symptoms	4.11 (3.80–4.46)	4.23 (3.88–4.61)	3.27 (2.65–4.02)	0.04
Temperature (°C)	37.02 (36.94–37.10)	36.98 (36.91–37.06)	37.32 (37.03–37.62)	0.03
Location of cellulitis				
Lower extremities	453 (74.8%)	403 (74.5%)	50 (76.9%)	0.9
Upper extremities	82 (13.5%)	74 (13.7%)	8 (12.3%)	
Thorax/abdomen	26 (4.3%)	24 (4.4%)	2 (3.1%)	
Head/neck	45 (7.4%)	40 (7.4%)	5 (7.7%)	
Exclusive or preferential side				
Right	264 (43.6%)	235 (43.4%)	29 (44.6%)	0.5
Left	292 (48.2%)	259 (47.9%)	33 (50.8%)	
Bilateral/centered	50 (8.3%)	47 (8.7%)	3 (4.6%)	
Maximum length of cellulitis (cm) ^b^				
(*n* = 398)	20.08 (18.76–21.50)	19.48 (18.13–20.93)	24.85 (20.14–30.67)	0.02
Crepitation				
Yes	9 (1.5%)	6 (1.1%)	3 (4.6%)	0.06
No	597 (98.5%)	535 (98.9%)	62 (95.4%)	
Presence of purulent collection				
Yes	164 (27.1%)	142 (26.2%)	22 (33.8%)	0.19
No / not detected	442 (72.9%)	399 (73.8%)	43 (66.2%)	
Detection of the collection				
By physical exam	104 (63.4%)	91 (64.1%)	13 (59.1%)	0.6
Only by imaging	60 (36.6%)	51 (35.9%)	9 (40.9%)	
Laboratory parameters				
Blood glucose (mg/dL)	124.4 (120.9–128.0)	123.3 (119.6–127.2)	133.6 (123.5–144.5)	0.09
Blood creatinine (mg/dL)	1.03 (1.00–1.07)	1.01 (0.97–1.04)	1.28 (1.11–1.47)	0.001
Leukocyte count (cells/μL)	10,777 (10354–11,218)	10,395 (9983–10,824)	14,555 (12575–16,847)	< 0.0001
Neutrophil count (% of leukocytes)	75.13 (73.95–76.34)	74.18 (72.92–75.47)	83.55 (81.31–85.86)	< 0.0001
ESR (mm/h)				
(*n* = 161)	53.02 (47.64–59.01))	52.84 (47.06–59.32)	54.59 (41.05–72.59)	0.8
CRP (mg/L)				
(*n* = 581)	23.60 (20.47–27.21)	22.06 (18.99–25.63)	41.04 (26.52–63.49)	0.008
Imaging procedures				
Imaging				
Yes	277 (45.7%)	247 (45.7%)	30 (46.2%)	0.9
No	329 (54.3%)	294 (54.3%)	35 (53.8%)	
Imaging ^a^				
Only echography	147 (53.1%)	136 (55.1%)	11 (36.7%)	0.2
Only CT	50 (18.1%)	44 (17.8%)	6 (20.0%)	
Only MRI	18 (6.5%)	15 (6.1%)	3 (10.0%)	
Others / combined	62 (22.4%)	52 (21.1%)	10 (33.3%)	
Imaging (single or combined) ^a^				
Echography	180 (65.0%)	160 (64.8%)	20 (66.7%)	0.8
Other than echo	97 (35.0%)	87 (35.2%)	10 (33.3%)	
CT	77 (27.8%)	65 (26.3%)	12 (40.0%)	0.11
Other than CT	200 (72.2%)	182 (73.7%)	18 (60.0%)	
MRI	37 (13.3%)	30 (12.1%)	7 (23.3%)	0.9
Other than MRI	240 (86.6%)	217 (87.9%)	23 (76.7%)	
Hospitalization parameters & outcome				
Days of hospital stay	7.02 (6.63–7.44)	6.88 (6.49–7.30)	8.30 (6.72–10.25)	0.09
Follow-up after discharge				
Primary care	377 (63.7%)	345 (64.6%)	32 (55.2%)	0.3
Outpatient clinic	202 (34.1%)	177 (33.1%)	25 (43.1%)	
Others	13 (2.2%)	12 (2.2%)	1 (1.7%)	
Cellulitis outcome				
Good response	520 (85.8%)	469 (86.7%)	51 (78.5%)	0.07
Poor response	86 (14.2%)	72 (13.3%)	14 (21.5%)	
Vital outcome				
Death	18 (3.0%)	11 (2.0%)	7 (10.8%)	0.001
Survival	588 (97.0%)	530 (98.0%)	58 (89.2%)	
Death related to cellulitis				
Yes	5 (27.8%)	1 (9.1%)	4 (57.1%)	0.047
No	13 (72.2%)	10 (90.9%)	3 (42.9%)	
Days from admission to death	7.18 (4.14–12.45)	9.05 (4.85–16.88)	5.16 (1.54–17.23)	0.3

Values are expressed as mean (95% CI) or % as appropriate

^a^ Respect to patients who underwent imaging procedures

No differences in imaging procedures were observed in cellulitis patients with or without sepsis. Regarding outcome, death was more common in patients with sepsis than in patients without this condition (*P* = 0.001). In addition, the cause of death was related to cellulitis more frequently in patients with sepsis compared to the remaining patients (*P* = 0.047). Septic shock developed in 5 of the 65 patients with sepsis (7.7%) and 3 of them died.

Table 3 shows the microbiological aspects of cellulitis patients with and without sepsis. Drawing of blood (*P* < 0.0001) or any (*P* = 0.0001) culture, and identification of the microbiological agent (*P* = 0.005) were more likely among patients with sepsis compared to non-septic individuals. Methicillin-resistant *Staphylococcus aureus* (MRSA) represented 24.6% of all isolates of *S. aureus* in either blood or pus, and 9.8% of all patients with positive cultures. Two patients with MRSA infection evolved to sepsis (13.3% of all MRSA infections) and both survived.

**Table 3:** Microbiological aspects associated with sepsis in patients with cellulitis

	All ***n*** = 606	No sepsis (***n*** = 541)	Sepsis (***n*** = 65)	***P*** value
Culture of the purulent collection				
Pus culture available				
Yes	150 (24.8%)	129 (23.8%)	21 (32.3%)	0.14
No	456 (75.2%)	412 (76.2%)	44 (67.7%)	
Results of culture				
Positive	118 (78.7%)	101 (78.3%)	17 (81.0%)	0.8
Negative	32 (21.3%)	28 (21.7%)	4 (19.0%)	
Positive culture				
Monomicrobial	92 (78.0%)	78 (77.2%)	14 (82.4%)	0.7
Polymicrobial	26 (22.0%)	23 (22.8%)	3 (17.6%)	
Aerobes (monomicrobial) *				
None	32 (21.3%)	28 (21.7%)	4 (19.0%)	0.6
*S aureus* only	45 (30.0%)	41 (31.8%)	4 (19.0%)	
Streptococci only	18 (12.0%)	14 (10.9%)	4 (19.0%)	
Gram-neg bacilli only	21 (14.0%)	17 (13.2%)	4 (19.0%)	
Others/polymicrobial	34 (22.7%)	29 (22.5%)	5 (23.8%)	
Aerobes (mono or polymicrobial) *				
*S aureus*	56 (37.3%)	51 (39.5%)	5 (23.8%)	0.17
No *S aureus*	94 (62.7%)	78 (60.5%)	16 (76.2%)	
Streptococci	24 (16.0%)	20 (15.5%)	4 (19.0%)	0.7
No streptococci	126 (84.0%)	109 (84.5%)	17 (81.0%)	
Gram-negative bacilli	43 (28.7%)	37 (28.7%)	6 (28.6%)	1
No Gram-neg bacilli	107 (71.3%)	92 (71.3%)	15 (71.4%)	
Anaerobes *				
Yes	8 (5.3%)	6 (4.7%)	2 (9.5%)	0.3
No	142 (94.7%)	123 (95.3%)	19 (90.5%)	
Blood culture				
Blood culture available				
Yes	252 (41.6%)	207 (38.3%)	45 (69.2%)	< 0.0001
No	354 (58.4%)	334 (61.7%)	20 (30.8%)	
Results of culture				
Positive	46 (18.3%)	35 (16.9%)	11 (24.4%)	0.2
Negative	206 (81.7%)	172 (83.1%)	34 (75.6%)	
Positive culture				
Monomicrobial	46 (100%)	35 (100%)	11 (100%)	–
Polymicrobial	0 (0.0%)	0 (0.0%)	0 (0.0%)	
Aerobes **				
None	206 (81.7%)	172 (83.1%)	34 (75.6%)	0.7
*S aureus*	8 (3.2%)	7 (3.4%)	1 (2.2%)	
Streptococci	18 (7.1%)	13 (6.3%)	5 (11.1%)	
Gram-neg bacilli	7 (2.8%)	5 (2.4%)	2 (4.4%)	
Others	13 (5.2%)	10 (4.8%)	3 (6.7%)	
Anaerobes				
Yes	1 (0.4%)	0 (0.0%)	1 (2.2%)	0.18
No	251 (99.6%)	207 (100%)	44 (97.8%)	
Pus or blood culture				
Any culture				
Yes	333 (55.0%)	282 (47.9%)	51 (78.5%)	0.0001
No	273 (45.0%)	259 (47.9%)	14 (21.5%)	
Any microorganism identified				
Yes	155 (25.6%)	129 (23.8%)	26 (40.0%)	0.005
No	451 (74.4%)	412 (76.2%)	39 (60.0%)	
Specific microorganisms ***				
None	178 (53.5%)	153 (54.3%)	25 (49.0%)	0.4
*S aureus* only	50 (15.0%)	45 (16.0%)	5 (9.8%)	
Streptococci only	33 (9.9%)	25 (8.9%)	8 (15.7%)	
Gram-neg bacilli only	28 (8.4%)	22 (7.8%)	6 (11.8%)	
Others/polymicrobial	44 (13.2%)	37 (13.1%)	7 (13.7%)	
Aerobes (mono or polymicrobial) ***				
*S aureus*	61 (18.3%)	55 (19.5%)	6 (11.8%)	0.19
No *S aureus*	272 (81.7%)	227 (80.5%)	45 (88.2%)	
Streptococci	40 (12.0%)	31 (11.0%)	9 (17.6%)	0.18
No streptococci	293 (88.0%)	251 (89.0%)	42 (82.4%)	
Gram-negative bacilli	50 (15.0%)	42 (14.9%)	8 (15.7%)	0.9
No Gram-neg bacilli	283 (85.0%)	240 (85.1%)	43 (84.3%)	
Causative microorganisms identified				
Yes	143 (23.6%)	120 (22.2%)	23 (35.4%)	0.006
No	453 (74.8%)	414 (76.5%)	39 (60.0%)	
Doubtful	10 (1.7%)	7 (1.3%)	3 (4.6%)	

* Respect to all patients with pus (*), blood (**) or any (***) culture

Table 4 shows the treatment of cellulitis patients with and without sepsis. Regarding therapy, patients with sepsis underwent changes in their initial antimicrobial regimen more commonly (*P* < 0.0001), received more antimicrobials (*P* < 0.0001), longer intravenous treatment (*P* = 0.03) and underwent surgery more frequently (*P* = 0.01) compared to patients without sepsis.

**Table 4:** Treatment characteristics associated with sepsis in patients with cellulitis

	All ***n*** = 606	No sepsis (***n*** = 541)	Sepsis (***n*** = 65)	***P*** value
Treatment before admission				
Yes	237 (39.1%)	218 (40.3%)	19 (29.2%)	0.08
No	369 (60.9%)	323 (59.7%)	46 (70.8%)	
Initial treatment at admission				
Single antibiotic	381 (62.9%)	347 (64.1%)	34 (52.3%)	0.06
> 1 antibiotic	225 (37.1%)	194 (35.9%)	31 (47.7%)	
Amoxicillin-clavulanate monotherapy				
Yes	259 (42.7%)	231 (42.7%)	28 (43.1%)	1
No	347 (57.3%)	310 (57.3%)	37 (56.9%)	
Change of the initial regimen				
Yes	184 (30.4%)	148 (27.4%)	36 (55.4%)	< 0.0001
No	422 (69.6%)	393 (72.6%)	29 (44.6%)	
Reason for change ^a^				
Culture	57 (31.0%)	46 (31.1%)	11 (30.6%)	0.3
Poor response	55 (29.9%)	40 (27.0%)	15 (41.7%)	
Toxicity	11 (6.0%)	9 (6.1%)	2 (5.6%)	
Others	61 (33.2%)	53 (35.8%)	8 (22.2%)	
Days until change ^a^	3.49 (3.16–3.85)	3.40 (3.05–3.79)	3.86 (3.02–4.94)	0.3
Treatment after change ^a^				
Single antibiotic	86 (46.7%)	71 (48.0%)	15 (41.7%)	0.5
> 1 antibiotic	98 (53.3%)	77 (52.0%)	21 (58.3%)	
Antibiotic treatment after discharge				
Yes	504 (85.1%)	453 (84.8%)	51 (87.9%)	0.5
No	88 (14.9%)	81 (15.2%)	7 (12.1%)	
Treatment after discharge				
Single antibiotic	407 (80.8%)	368 (81.2%)	39 (76.5%)	0.4
> 1 antibiotic	97 (19.2%)	85 (18.8%)	12 (23.5%)	
Total number of antibiotics used	1.59 (1.53–1.65)	1.55 (1.49–1.61)	2.02 (1.78–2.28)	< 0.0001
Total number of antibiotics used				
1	289 (47.7%)	271 (50.1%)	18 (27.7%)	0.0002
2	191 (31.5%)	169 (31.2%)	22 (33.8%)	
3	89 (14.7%)	74 (13.7%)	15 (23.1%)	
4	35 (5.8%)	25 (4.6%)	10 (15.4%)	
5	2 (0.3%)	2 (0.4%)	0 (0.0%)	
Days of IV antibiotic treatment	6.14 (5.78–6.52)	6.00 (5.63–6.38)	7.48 (6.05–9.26)	0.03
Total days of antibiotic treatment	13.27 (12.70–13.87)	13.11 (12.54–13.71)	14.72 (12.39–17.50)	0.19
Surgical treatment				
Yes	81 (13.4%)	66 (12.2%)	15 (23.1%)	0.01
No	525 (86.6%)	475 (87.8%)	50 (76.9%)	

Values are expressed as mean (95% CI) or % as appropriate

^a^ Only in patients who underwent treatment modification respect to the initial regimen

A logistic regression model was constructed using the variables with a *P* value < 0.1 in the univariate analysis, excluding death, to identify the factors independently associated with sepsis (Table 5). Increased blood leukocytes (*P* = 0.002) and serum creatinine (*P* = 0.003), blood culture drawn (*P* = 0.004), modification of the initial antimicrobial regimen (*P* = 0.007) and greater extent of the cellulitis area (*P* = 0.009) were independently associated with sepsis development in the multivariate analysis. As this model included parameters dependent, in different degrees, on the clinicians’ decisions (drawing of cultures, changes in therapy, etc.), the analysis was repeated after excluding such parameters. As expected, the results were very similar, with the only substitution of neutrophil counts, expressed as percent of leukocytes, by the total leukocyte counts (now *P* = 0.07). Thus the factors independently associated with sepsis were: serum creatinine (OR 2.335 [95% CI 1.452–3.755], *P* = 0.0005), extent of cellulitis (OR 1.027 [1.008–1.046], *P* = 0.005) and neutrophil count (OR 1.052 [1.009–1.097], *P* = 0.02).

**Table 5:** Variables independently associated with sepsis in patients with cellulitis

	OR (95% CI)	P
Leukocyte counts (per 1000 cells/μL)	1.080 (1.026–1.137)	0.003
Serum creatinine (per mg/dL)	2.238 (1.378–3.637)	0.001
Blood culture available	2.984 (1.443–6.169)	0.003
Modification of the initial antibiotic regimen	2.154–1.068-4.347)	0.03
Maximum length of cellulitis (per cm)	1.029 (1.009–1.049)	0.004

## Discussion

To our knowledge this is the first study devoted to analyze the factors related to the development of sepsis in patients with cellulitis. In our large series, blood cultures drawing, modification of the initial antimicrobial regimen, extension of cellulitis and increased blood leukocytes and creatinine were associated with this complication.

Sepsis was diagnosed in 10.7% of the hospitalized patients with cellulitis in our study, a rate very similar to the 10.77% of “bacteremia/endocarditis/septicemia/sepsis” cases reported for the subset of “abscess/cellulitis” in a study using a large administrative database [1]. On the contrary, our sepsis rate was higher than the 1.6% overall (5.3% considering only patients with bacteremia) reported in a Spanish retrospective study of 308 patients from 1997 to 2004 with limb cellulitis and similar comorbidities as our patients [9]. However, it should be taken into account that the authors consider only cases of “severe sepsis” according to the 1992 definition [23], a denomination that was considered superfluous in the 2016 update [24]. Similarly, another study involving multiple types of SSTI, including the most severe forms, found that severe sepsis developed in 9.5% of their patients, and septic shock in 3.9% [25]. Likewise, other authors reported that 15 out of 332 patients (4.5%) presented with “shock”, although no information about the origin was provided [8].

Diabetes has been considered a risk factor for bacteremia in cellulitis [10, 11, 18]. Among our diabetic patients 14.4% developed sepsis, a figure similar to the 16.9% reported in a large repository database using ICD codes for the subset of diabetics hospitalized with cellulitis/abscess who developed bacteremia/endocarditis/sepsis within the 0–64 age range [18]. Lymphedema has also been considered a predisposing factor for bacteremia [14, 20, 22]. However, 10.1% of the patients with edema/lymphedema in our series developed sepsis, a figure similar to the 10.7% observed in the patients as a whole. Recurrent episodes of cellulitis or the existence of wounds or skin lesions were not associated with sepsis in our study.

The bacteremia rate in our cohort was 18.3% among the patients with blood culture drawn, a value situated in the high range of those previously reported in cellulitis [4–16], although rates of 18.7% [8] and 21.3% [9] have been reported in hospitalized patients like ours. Of note is that blood cultures were positive in only 24.4% of patients with sepsis. Seemingly, prior antibiotic therapy played a major role in this low positivity rate. Thus, many patients were already receiving antibiotics at the time of arrival at the hospital and others, who did not present with sepsis but developed this complication later, may not have undergone blood culture initially, but later when their clinical condition worsened, while were receiving antibiotic therapy. Additional explanations might be untimely blood sampling in patients with intermittent bacteremia or inadequate blood culture drawing or processing. Anyhow sepsis development independently associated with drawing of blood for culture.

All positive blood cultures were monomicrobial, and streptococci were the most commonly isolated pathogen from blood (7.1% of all blood cultures and 11.1% among patients with sepsis). Our results agree with most reports that describe streptococci as the most commonly isolated microorganism from blood cultures in SSTI [5–10, 19].

Overall, the causative microorganism was identified, either in pus or blood, in about one-fourth of the patients in our study, reaching 40.0% in patients with sepsis. Community-acquired MRSA infection rate is increasing, particularly in SSTI. A study from Hawaii, one of the world places with higher MRSA prevalence, reported 62% of MRSA isolates in patients with abscesses or skin ulcers [26], data from the USA points out to figures of at least 50% [22, 27, 28], and this agent was isolated in 13–22% of patients from Spain [25, 29]. In our multicenter series, MRSA was identified in 24.6% of all pure or mixed isolates of *S. aureus* in either blood or pus (9.8% of all patients with positive cultures). Although the absolute number of MRSA infections in our series is too low to draw definite conclusions, MRSA does not seem to be more prone to the development of sepsis than other agents.

Not unexpectedly, sepsis associated with mortality in our study. In fact, the mortality rates, both overall and those caused by the infection, were five-fold higher in septic than in non-septic patients, reaching 10.8% overall and 57.1% when analyzing the deaths directly related to cellulitis. This mortality rate is consistent with the in-hospital mortality > 10% reported for sepsis and > 40% for septic shock by the Third International Consensus Definitions for Sepsis and Septic Shock working group [24].

The lack of studies on sepsis in cellulitis precludes any comparison. However, a study focused on the comparison of cellulitis with necrotizing fasciitis in patients admitted to an intensive care unit reported an in-hospital or 30-day mortality rate as high as 56.5% (13/23 patients) for cellulitis and 64.5% (20/31 patients) for necrotizing fasciitis [3]. Another study reported an overall 30-day mortality of 4.8% in patients with cellulitis, and a mortality rate due to shock or multiorgan failure of 3.0% [8]. Five out of our 65 patients with sepsis (7.7%) developed septic shock, and 3 of them died. In the above mentioned study, septic shock developed in 30 and 61% of the patients with cellulitis or necrotizing fasciitis, respectively [3].

Sepsis was also associated with increased blood leukocyte counts, as expected in the setting of systemic inflammatory response syndrome (SIRS) [24]. It also associated with high serum creatinine levels, which seems be due to the renal dysfunction commonly observed in sepsis [24], although certain degree of previous renal insufficiency in some patients as a predisposing factor for cellulitis and/or sepsis cannot be completely ruled out.

Patients with sepsis received more intensive medical and surgical treatment than those not septic during hospitalization. Sepsis was also associated with modification of the initial antimicrobial regimen in the multivariate analysis, mainly due to poor response to the initial treatment. Therefore, it seems clear that the severity of the infection, and the unsatisfactory response obtained, led to the attending physicians to change the initial therapeutic regimen.

Finally, and interestingly, sepsis associated with the extent of the cellulitis area. Once again, the lack of studies on this topic precludes contrasting our observation with other experiences. However, a study on limb cellulitis found in the univariate analyses an increased risk of bacteremia in patients with extension to the whole limb, the proximal limb, and the trunk, although only proximal limb involvement remained predictive in the multivariate analysis [9]. This association between the extent of the infection and the risk of bacteremia and sepsis makes sense, and suggests that the dimension of the infectious area should be registered in the patient’s medical record, and that patients with large cellulitis areas should be particularly monitored for the development of this serious and potentially lethal complication.

The main strengths of our study are the large sample size, its prospective nature and the elevated number of parameters evaluated, which allowed to adjust for and minimize the effect of confounders. Limitations include the absence of a unified treatment protocol, which would allow the evaluation of the responses to the same treatment, and the variability characteristic of multicenter studies, particularly regarding procedural and management aspects. However, currently no single or widely accepted treatment protocol exists [3, 17, 19–21], considering the great diversity of clinical and microbiological issues related to cellulitis, and the multicenter character of the study allows the evaluation of the real clinical practice across our country, minimizing potential biases from a single institution.

## Conclusions

From our large study, the first carried out on this topic, we conclude that blood culture drawing and modification of the initial antimicrobial regimen were more frequently observed in patients who developed sepsis. Similarly, blood leukocytes, creatinine, and the extent of cellulitis were also independently associated with sepsis development, and these three easily available parameters might be useful to identify those patients prone to develop sepsis and to optimize their management.

## Data Availability

The datasets generated and analysed during the current study are not publicly available due to privacy but are available from the corresponding author on reasonable request.

## Abbreviations

CI: Confidence interval
CRP: C-reactive protein
HUCA: Hospital Universitario Central de Asturias
IDSA: Infectious Diseases Society of America
MRSA: Methicillin-resistant *Staphylococcus aureus*
SIRS: Systemic inflammatory response syndrome
SSTI: Skin and soft tissue infection
